# Public health round-up

**DOI:** 10.2471/BLT.26.010526

**Published:** 2026-05-01

**African road safety framework**

The African Road Safety Charter entered into force on 12 March 2026. The charter marks Africa’s first continental and legally-binding road safety framework. Mozambique’s ratification in February made it the fifteenth required African Union Member State to trigger implementation. The WHO African Region faces nearly 250 000 road deaths annually, the highest rate globally. The charter compels signatories to align with the *Global plan for the decade of action for road safety 2021–2030* and the *African road safety action plan 2021–2030* which offer guidance to meet the goal of halving road deaths and serious injuries by 2030.

**Figure:**
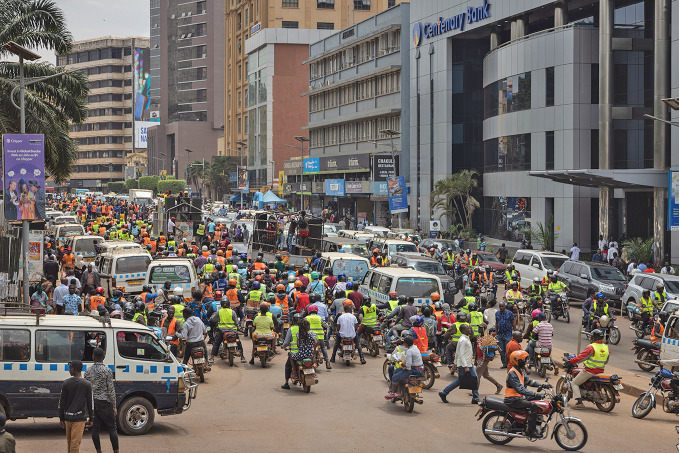
WHO/Esther Ruth Mbabazi

## Strengthening One Health

The Fourth Quadripartite Executive Annual Meeting took place in Lyon, France, on 8–9 April 2026, bringing together the Food and Agriculture Organization, the United Nations Environment Programme, the World Health Organization (WHO) and the World Organisation for Animal Health to advance the One Health approach. 

As global threats such as emerging infectious diseases, antimicrobial resistance, climate change and environmental degradation intensify, the meeting focused on strengthening coordinated systems linking human, animal, plant and ecosystem health. 

Building on the *One Health joint plan of action*, the quadripartite advanced progress across four priority areas: supporting countries in implementing One Health through stronger multisectoral coordination and national planning; improving scientific collaboration, integrated data systems and evidence-sharing; enhancing governance and policy frameworks to embed One Health across sectors; and mobilizing sustainable financing to scale up action. 

These efforts aimed to help countries prevent risks at source, detect threats early and respond effectively, while building resilient food systems, healthy ecosystems and robust veterinary and public health services. The quadripartite called on governments, financial institutions, development partners and the private sector to invest in integrated approaches and long-term capacity, reaffirming its commitment to coordinated global action that strengthens resilience at the human–animal–environment interface.

https://bit.ly/4mF3IxP

## Global forum of collaborating centres 

WHO convened the first Global forum of collaborating centres, uniting representatives from more than 800 institutions across over 80 countries in one of the world’s largest public health networks. The meeting highlighted the growing health threats emerging in an increasingly fragmented world and underscored the need for stronger scientific cooperation. Participants reaffirmed their commitment to shifting from isolated scientific projects towards more integrated, dynamic partnerships. 

The collaborating centres network, rooted in WHO’s constitutional mandate since 1949, has expanded over 77 years into a global system of leading academic, research and technical institutions that support innovation, standards-setting and capacity-building.

Aligned with the international One Health Summit and the World Health Day 2026 theme “Together for health. Stand with science”, the forum emphasized the essential role of science in addressing complex global health challenges and stressed that strong international cooperation and sustained investment remain vital, particularly amid declining global health financing.

“Science is at the heart of everything we do to protect and improve health,” said Sylvie Briand, WHO Chief Scientist. “The global network of WHO collaborating centres represents an extraordinary concentration of scientific expertise and public health leadership. Together, they form a powerful force for knowledge, innovation and action. At a time of growing global health challenges, this spirit of trusted scientific collaboration is not only valuable, it is indispensable to protecting lives and shaping a healthier future for all.”

WHO also announced the expansion of its collaborating centres through new collaborative open research consortia aimed at accelerating research on vaccines, diagnostics and treatments for disease X. 

https://bit.ly/4vAROcF

## Three years of conflict in Sudan

After three years of conflict, Sudan has become the world’s largest humanitarian and health crisis, with 34 million people needing assistance and 21 million lacking access to medical services. Continued fighting has devastated an already fragile health system, driving sharp rises in disease outbreaks and malnutrition while funding and access decline. “The war in Sudan is devastating lives and denying people their most basic rights, including health, water, food and safety,” said WHO Director-General Tedros Adhanom Ghebreyesus. “Doctors and health workers can save lives, but they must have safe places to work and the medicines and supplies they need. Ultimately, the best medicine is peace.”

More than 4 million people are acutely malnourished, heightening vulnerability to illness. Outbreaks of malaria, dengue, measles, polio, hepatitis E, meningitis and diphtheria have been reported across multiple states. Nationwide, 37% of health facilities are non-functional and WHO has verified 217 attacks on health care since April 2023, resulting in 2052 deaths and 810 injuries. 

WHO has strengthened Sudan’s health system by restoring key public health services, supporting laboratories and maintaining the supply chain for essential medicines and equipment. Since April 2023, it has delivered more than 3300 metric tons of medical supplies for cholera, malaria, nutrition and trauma care. WHO-supported clinics, hospitals and mobile teams have provided care to over 4.1 million people, treated 118 000 children with severe acute malnutrition, and reached more than 46 million people through vaccination campaigns.

 “With millions lacking basic medical care, facing hunger and at risk of disease, Sudan’s health crisis continues to deepen, emphasizing the urgent need for humanitarian support and long-term solutions. We remain committed to the people of Sudan,” said WHO Regional Director for the Eastern Mediterranean, Hanan Balkhy.

https://bit.ly/4mH3G8L

## Chile eliminates leprosy 

Chile has been officially verified by WHO as having eliminated leprosy, marking a historic milestone for regional public health. The achievement follows more than 30 years without a locally acquired case. 

An independent expert panel convened by WHO and the Pan American Health Organization (PAHO) in 2025 confirmed the absence of local transmission and validated Chile’s capacity to detect and manage imported cases. Between 2012 and 2023, the country reported 47 cases, all acquired abroad.

“Chile’s achievement demonstrates that eliminating leprosy is achievable and requires building strong systems that can detect, respond to, and provide comprehensive care for people affected by the disease, including those living with chronic disabilities,” said PAHO Director Jarbas Barbosa. “Being the first country in the Americas to be confirmed as eliminating leprosy sends a powerful message to the Region, that diseases strongly linked to groups living in vulnerable conditions can be eliminated, contributing to interrupt the vicious circle between disease and poverty.”

Health Minister Ximena Aguilera called the verification “a source of great pride,” crediting decades of prevention, early diagnosis, treatment and stigma‑free care. Chile’s model, built on strong surveillance, trained clinicians, rehabilitation services and legal protections for affected people, now serves as a blueprint for other nations in the Americas striving towards disease elimination.

https://bit.ly/4theGfv

## Health of migrants and refugees 

More than 60 countries now include refugees and migrants in their national health policies and laws, marking what the World Health Organization describes as a major global shift towards more inclusive health systems. The findings come from WHO’s first‑ever global baseline report on migrant‑responsive health systems, drawing on data from 93 Member States.

Today, over one billion people live as refugees or migrants. Many face significant barriers to care, along with heightened risks of infectious diseases, chronic conditions, mental‑health challenges and unsafe living or working environments.

“Refugees and migrants are not just recipients of care, they are also health workers, caregivers and community leaders,” said WHO Director‑General Tedros Adhanom Ghebreyesus. “Health systems are only truly universal when they serve everyone.”

The new *World report on promoting the health of refugees and migrants: monitoring progress on the WHO global action plan* shows that even in politically sensitive contexts, countries are increasingly relying on evidence and established norms and standards to guide how migration and health are addressed within national health systems. 

Despite progress, gaps persist. Only 37% of countries routinely collect migration‑related health data, and fewer than 40% train health workers in culturally responsive care. Refugees generally access services more easily than irregular migrants, internally displaced people or migrant workers, who remain inconsistently covered. Participation in health governance also remains limited, underscoring the need for more inclusive approaches as countries strengthen preparedness for future health challenges. 

https://bit.ly/48bwLmU

**Cover photo**

Children and their mothers stand holding food baskets with nutrient‑rich options encouraged for families for a balanced and varied diet, Antsakoantsoa Centre community site, Tsihombe district, Androy region, Madagascar.

**Figure:**
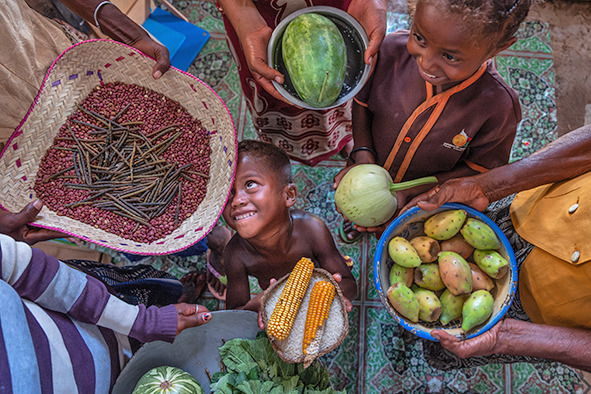
UNICEF/Andrianantenaina

**Looking ahead**

5–8 May. Global Pre-eclampsia Summit. Kigali, Rwanda. https://bit.ly/4euvcUS

18–23 May. Seventy-ninth World Health Assembly. Geneva, Switzerland. https://bit.ly/4egtTZV

31 May. World No Tobacco Day. Global events https://bit.ly/4mEXkXD

